# Role of apoptosis-inducing factor (AIF) in programmed nuclear death during conjugation in *Tetrahymena thermophila*

**DOI:** 10.1186/1471-2121-11-13

**Published:** 2010-02-11

**Authors:** Takahiko Akematsu, Hiroshi Endoh

**Affiliations:** 1 Division of Life Science, Graduate School of Natural Science and Technology, Kanazawa University, Shizenken, Kakuma-machi, Kanazawa, Ishikawa, Japan

## Abstract

**Background:**

Programmed nuclear death (PND), which is also referred to as nuclear apoptosis, is a remarkable process that occurs in ciliates during sexual reproduction (conjugation). In *Tetrahymena thermophila*, when the new macronucleus differentiates, the parental macronucleus is selectively eliminated from the cytoplasm of the progeny, concomitant with apoptotic nuclear events. However, the molecular mechanisms underlying these events are not well understood. The parental macronucleus is engulfed by a large autophagosome, which contains numerous mitochondria that have lost their membrane potential. In animals, mitochondrial depolarization precedes apoptotic cell death, which involves DNA fragmentation and subsequent nuclear degradation.

**Results:**

We focused on the role of mitochondrial apoptosis-inducing factor (AIF) during PND in *Tetrahymena*. The disruption of *AIF *delays the normal progression of PND, specifically, nuclear condensation and kilobase-size DNA fragmentation. AIF is localized in *Tetrahymena *mitochondria and is released into the macronucleus prior to nuclear condensation. In addition, AIF associates and co-operates with the mitochondrial DNase to facilitate the degradation of kilobase-size DNA, which is followed by oligonucleosome-size DNA laddering.

**Conclusions:**

Our results suggest that *Tetrahymena *AIF plays an important role in the degradation of DNA at an early stage of PND, which supports the notion that the mitochondrion-initiated apoptotic DNA degradation pathway is widely conserved among eukaryotes.

## Background

Among protists, ciliates have evolved complicated structures for the spatial segregation of the germline and soma, irrespective of their unicellular organization. One remarkable feature of ciliates is their nuclear dualism. Ciliates bear two functionally and morphologically distinct nuclei within the same cytoplasm: a reproductive somatic macronucleus and a germinal micronucleus. The polyploid macronucleus is large and supports almost all vegetative functions through active transcription, whereas the diploid micronucleus is transcriptionally silent [1]. These nuclei both originate from a fertilized micronucleus (synkaryon) via two successive postzygotic divisions (PZDs) during a unique form of sexual reproduction known as conjugation. Programmed nuclear death (PND), also known as nuclear apoptosis, is a unique process in ciliates whereby only the parental macronucleus is eliminated from the cytoplasm of the progeny during conjugation, while the parental cytoplasm is taken over by the progeny, even after sexual reproduction. In *Tetrahymena thermophila*, once the new macronucleus differentiates from the synkaryon, the parental macronucleus begins to degenerate. This degeneration has three distinct stages, beginning with the degeneration of the macronuclear DNA into large (> 30-kb) fragments. This fragmentation occurs prior to nuclear condensation and involves Ca^2+^-independent, Zn^2+^-insensitive nuclease activity [2]. In the second stage, marked changes occur in the degenerating macronucleus, including size reduction and chromatin condensation. During this second stage, the macronuclear DNA is degraded into smaller fragments, which comprise an oligonucleosome-scale ladder that consists of ~180-bp units [3,4]. Meanwhile, many small autophagosomes approach and engulf the nucleus, resulting in the formation of a large autophagosome with a double membrane [5]. At this stage, lysosomes are closely associated with the autophagosome without fusion, indicating that the pH of the parental macronucleus is still neutral. In the third stage, the macronuclear DNA is degraded completely. Lysosomes fuse with the autophagosomal membrane, releasing their contents into and acidifying the macronucleus, which is then resorbed through autophagy in the acidic environment [6].

Kobayashi and Endoh [7] indicated that autophagosomes contain many mitochondria that have lost their membrane potential. In general, the loss of mitochondrial membrane potential leads to the release of cytochrome c and apoptosis-inducing factor (AIF) into the cytosol [8]. Thus, it is reasonable to assume that the mitochondrial pathway plays a key role in *Tetrahymena *PND. Indeed, mitochondria play key roles in a number of apoptotic and programmed cell death (PCD) processes in animals, such as morphogenesis, tissue homeostasis, and immunity [9]. In animals, apoptosis involves both caspase-dependent and caspase-independent pathways. Cytochrome c participates in the activation of caspases, which are major effectors of apoptosis, whereas AIF is involved in the caspase-independent pathway [10,11]. Caspase activation affects a number of substrates with important biological functions, leading to the loss of their functional roles [12]. However, it is unclear whether PCD in plants and protozoa involves the activation of caspase-like enzymes. Considering that caspase homologs are not present in fungi, plants, and protists, with the exception of animals [13], the origins of these activities remain unknown. Furthermore, isolated mitochondria from *T. thermophila *show strong DNase activity, similar to that of human endonuclease G (EndoG), which mediates the caspase-independent apoptotic pathway (also referred to as mitochondrial pathway) [7]. Based on these information, PND looks to occur by the caspase-independent pathway. However, an EndoG homolog has not been identified in the *Tetrahymena *genome database.

AIF is a nuclear-encoded mitochondrial flavoprotein that possesses NADH oxidase activity in its C-terminal region. The primary sequence of AIF is highly homologous to those of oxidoreductases from animals, fungi, plants, eubacteria, and archaebacteria [13,14]. AIF is a novel, mammalian, caspase-independent death effector that, upon the induction of apoptosis, translocates from the mitochondrial intermembrane space to the nucleus [15]. Once in the nucleus, AIF causes chromatin condensation and large-scale (~50 kb) DNA fragmentation [8]. AIF-mediated PCD has been observed in roundworms (*Caenorhabditis elegans*) [16] and in a cellular slime mold (*Dictyostelium discoideum*) [17], which suggests that the AIF pathway is a phylogenetically primitive form of apoptosis.

In the present study, we investigated whether the pro-apoptotic function of AIF is conserved in *Tetrahymena *PND. To address this issue, we cloned the *Tetrahymena *AIF homolog and performed gene disruption to analyze its biological functions. We discuss the unique evolution of apoptotic mechanisms.

## Results

### PND in *T. thermophila*

**Figure 1:**
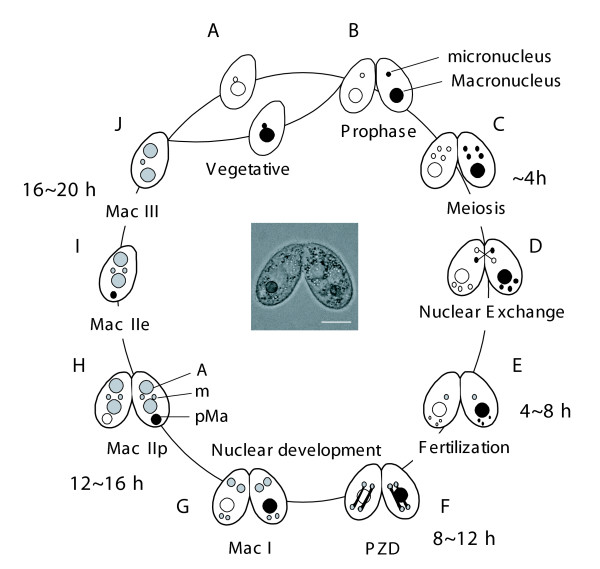
**Nuclear events during conjugation of *Tetrahymena thermophila***. Conjugation in *T. thermophila *is a complicated process that is initiated by interaction between complementary mating types, which form a conjugating pair. A. Vegetative phase. B. Meiotic prophase. C. Meiosis. D. Nuclear exchange. One of four meiotic products mitotically divides, forming two pronucei. Subsequently, one of the pronuclei is reciprocally exchanged between mating partners. E. Fertilization (synkaryon formation). F. PZD (postzygotic division). Fertilized nucleus successively divides twice. G. Mac I. Anteriorly-located nuclei differentiate into the new macronuclei, while posterior nuclei remain the micronuclei. H. Mac IIp. The parental macronucleus migrates posteriorly and begins to degenerate. I. Mac IIe. Pair separates (exconjugants). J. Mac III. One of two micronuclei is eliminated. Progeny of *T. thermophila *do not undergo conjugation during the first ~100 vegetative fissions after conjugation called "immature." A: macronuclear anlagen. m: micronuclei. pMa: parental macronucleus. The scale bar in photograph indicates 10 μm.

The nuclear events that are typical for conjugation in *T. thermophila *and that are specifically involved in nuclear apoptosis are illustrated schematically in Figure 1. Although previous studies have reported the details of these events [2,3], we show the timing of these events with regard to our experimental conditions.

### Identification of the *Tetrahymena *homolog of AIF

Using the *Tetrahymena *genome database http://www.ciliate.org/, we identified two AIF homologs (TTHERM_00622710 and TTHERM_01104910) that are similar to human AIF. As described below, one of these homologs, TTHERM_01104910, had no apparent effect on mitochondrial nuclease activity (Additional File 1). Therefore, in the present study, we focused on the role of the TTHERM_00622710 homolog.

**Figure 2:**
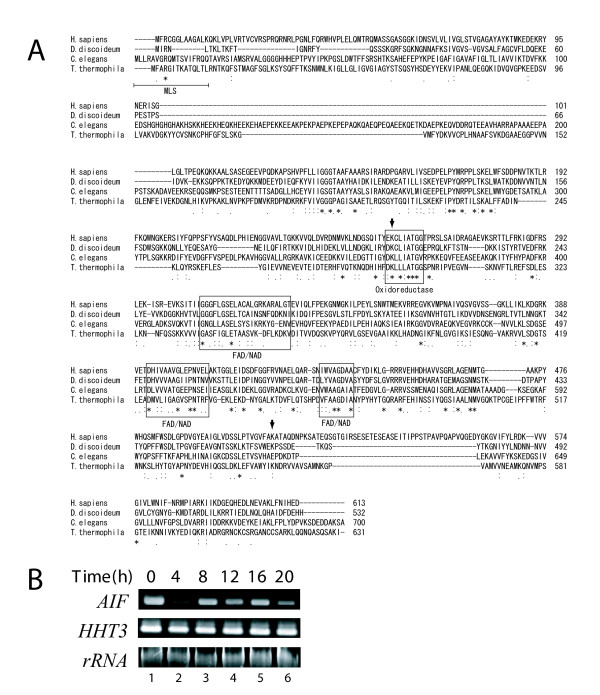
**Sequence alignment of AIF proteins and analysis of AIF expression**. A. CLUSTAL-W was used to generate AIF sequence alignment, including *Homo sapiens*, *Dictyostelium discoideum*, *Caenorhabditis elegans *and *Tetrahymena thermophila*. Boxes indicate the NAD/FAD binding domain and oxidoreductase domain. MLS in N-terminal portion of *T.thermohila *indicates mitochondrial localization sequence. Asterisks indicate identical amino acids. Colons and semicolons indicate amino acid similarity. Arrowheads indicate a potential DNA binding site of human AIF. B. RT-PCR analysis of *AIF *transcript during conjugation. *Histone h3 *(*HHT3*) was used as a control. SSU rRNA was used as a loading control.

This gene for *Tetrahymena *AIF lacks introns and encodes a 70-kDa protein. A primary sequence comparison revealed that residues in the FAD/NAD binding domain and the oxidoreductase domain of *Tetrahymena *AIF are highly conserved in human AIF (GenBank accession no. AAD16436.1), cellular slime mold AIF (GenBank; EAL63305.1), and the *C. elegans *AIF homolog Wah-1 (NCBI; NP_499564.2) (Figure 2A). *Tetrahymena *AIF is ~24% identical and 45% similar to human AIF. The putative DNA-binding sites in human AIF, which are required for its interaction with DNA and the induction of cell death [18], were identified in each phylum. MitoProt II, which is a prediction server for mitochondrial targeting sequences and cleavage sites, revealed a candidate mitochondrial localization sequence (MLS) in the N-terminus of *Tetrahymena *AIF (residues 1-13). However, the N-terminal portions of the remaining three proteins showed no sequence similarity.

To determine whether endogenous *Tetrahymena AIF *is constitutively expressed during conjugation, mRNA samples extracted from starved cells (just before mixing the mating types) and conjugating cells were subjected to RT-PCR analysis. Using *AIF*-specific primers, a single 340-bp band was detected in the starved cells (Figure 2B, lane 1). The mRNA of conjugating cells was extracted every 4 h (4 - 20 h) after the initiation of conjugation. *AIF *was expressed continuously during conjugation, although expression decreased at 4 h (Figure 2B, lane 2), which corresponded to the meiotic prophase. In the control experiment, *histone h3 *(*HHT3*) was also found to be expressed during conjugation as shown in previous study [19].

### AIF translocates from the mitochondria to the parental macronucleus

**Figure 3:**
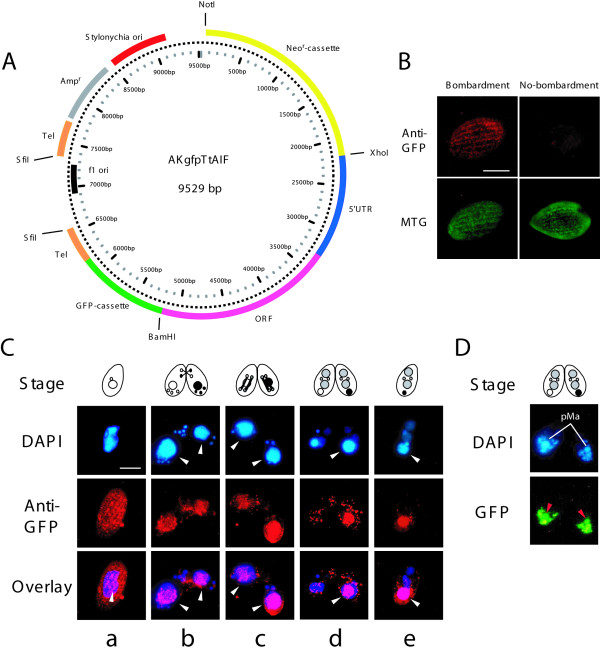
**Translocation of AIF from mitochondria to parental macronucleus**. A. Map of expression vector, named AKgfpTtAIF, with neomycin resistance cassette (Neo^r^-cassette), 5'UTR and ORF of *AIF*, codon-optimized GFP (GFP-cassette), replication origin derived from *Stylonychia lemnae *and telomeres from *Tetrahymena*. Neomycin resistance is expressed under control of *β-tubulin *promoter. Before biolistic bombardment, the plasmid was digested with SfiI to expose telomere sequences on both ends. B. After biolistic bombardment, cytoplasmic localization of AIF::GFP was detected using α-GFP. This fluorescent pattern was coincided with MitoTracker Green (MTG). No-bombardment indicates non-transformed wild-type cell. Scale bar in photograph indicates 10 μm. C. Translocation of AIF::GFP was visualized with α-GFP antibodies. White arrows indicate parental macronucleus. Overlay represents fusion image of blue (nuclei) and red (AIF::GFP) fluorescence. Scale bar in photograph indicates 10 μm. D. Fluorescence microscopy of living cells expressing AIF::GFP at the stage of MacIIp. Red arrows indicate AIF::GFP in parental macronuclei. pMa denotes parental macronucleus.

To examine the subcellular localization of *Tetrahymena *AIF, we constructed a plasmid that expresses a fusion protein composed of AIF and GFP (AIF::GFP) under the control of the *AIF *promoter (Figure 3A). The transformants stably expressed AIF::GFP in the presence of 50 μg/ml paromomycin, whereas paromomycin at concentrations >50 μg/ml had detrimental effects on cell growth. However, the signal was too weak to allow cytological analysis. To solve this problem, we performed indirect immunofluorescence using GFP-specific polyclonal antibodies, to determine the localization of the fusion protein. AIF::GFP signals were observed at the surface and in the cytoplasm of each cell (Figure 3B), similar to the pattern observed with MTG, a mitochondrion-specific fluorescent dye. Therefore, AIF is localized within mitochondria.

To examine the translocation of AIF during conjugation, the localization of the fusion protein (AIF::GFP) was observed throughout this process. As shown in Figure 3Ca, before conjugation, AIF::GFP was distributed over the cell surface along the ciliary rows. During nuclear exchange, the pattern of AIF expression changed, and intense signals were detected in the posterior region of each cell (Figure 3Cb). Meanwhile, the signals nearly overlapped (and probably surrounded) the parental macronucleus during the stages that correspond to PZD to Mac IIe (Figure 3Cc-e, see also Figure 1). Living cells expressed AIF::GFP during the MAC IIp stage (Figure 3D). Although the AIF::GFP signal in living cells was weak, as mentioned above, the signal was concentrated and visualized at the posterior region of the cell, where it overlapped with the parental macronucleus (indicated by red arrows in the Figure). These results suggest that AIF is released from mitochondria and translocates to the parental macronucleus before nuclear condensation.

### AIF plays roles in the growth and PND of *T. thermophila*

**Figure 4:**
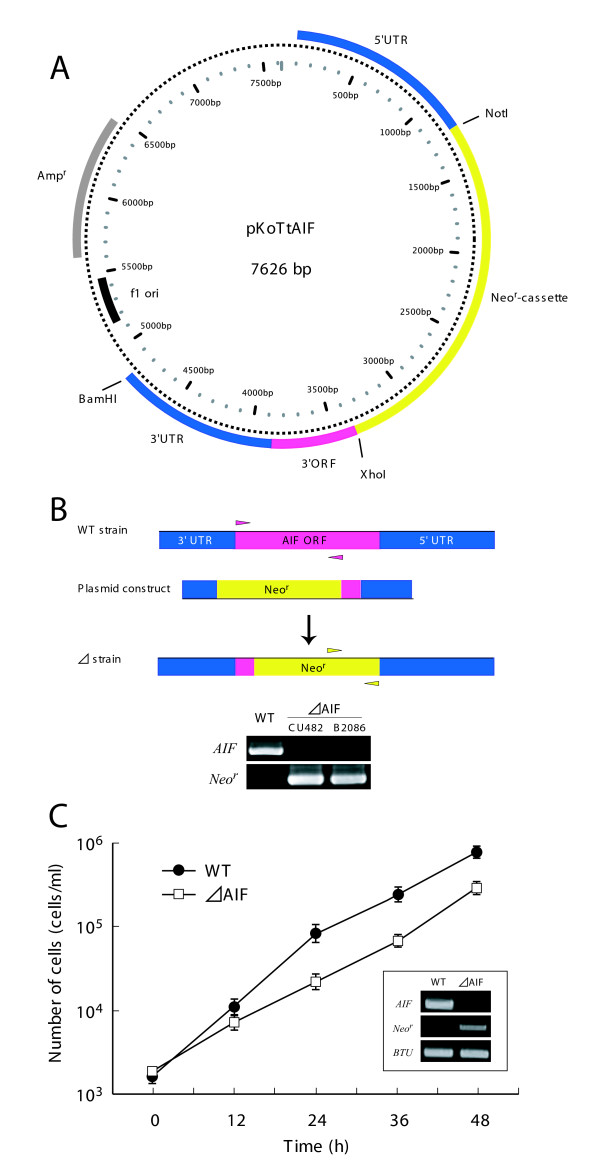
**Construction of *AIF*-deficient strain**. A. Map of expression vector, pKoTtAIF, with neomycin resistance cassette (Neo^r^-cassette). It consists of 5' and 3' UTR sequences of *AIF *and part of its 3' ORF. The neomycin resistance is expressed under the control of *β-tubulin *promoter. Before biolistic bombardment, the plasmid was digested with BamHI. B. Schematic representation of the wild-type (WT) and mutant locus of the *AIF *together with the targeting plasmid. Replacement of *AIF *to neomycin-resistant gene (*Neo*^*r*^) in the macronucleus was confirmed by PCR using 10 ng of genomic DNA from isolated macronuclei as template. Small triangles located in the gene loci indicate specific primer pairs for the PCR amplification. C. Cell growth curve of CU428. Circles and squares indicate cell density of wild-type strain and *ΔAIF*, respectively. Points and attached bars correspond to the means of four identical measurements and standard deviations. The inset indicates RT-PCR analysis of the expression levels of *AIF *and *Neo*^*r*^. *β-tubulin *(*BTU*) was used as a control.

**Figure 5:**
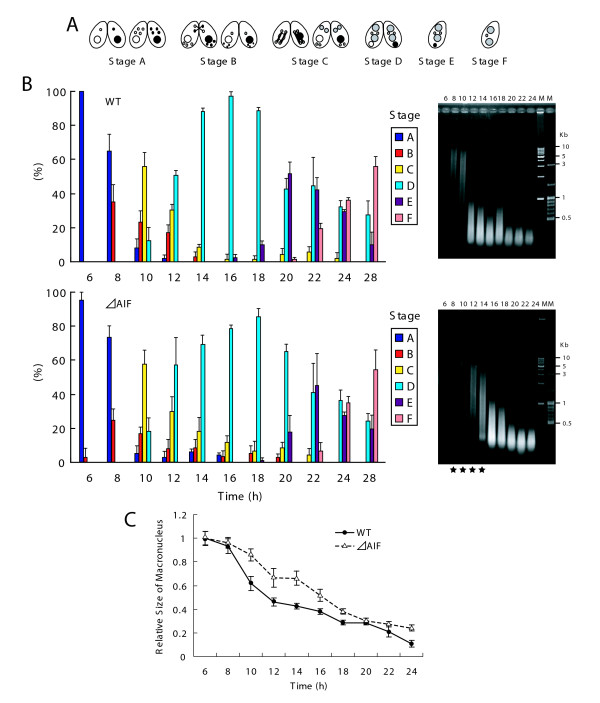
**Progression of PND by disruption of *AIF***. A. Nuclear events during conjugation were divided into 6 stages (stage A ~stage F). B. Time course analysis of progression of the nuclear events in wild-type and *ΔAIF *between 6 h and 28 h after initiation of conjugation. The percentages of the nuclear stages were counted, and were expressed as a percentage of the total number of tested cells (300-400 cells). Columns and attached bars correspond to the means of four identical measurements and standard deviations. Fragmental DNA was isolated from the strains every 2 h during conjugation. The black stars between 8-12 h in *ΔAIF *indicate delay of kb-sized DNA fragmentation. M denotes kbp-ladder size marker (left) and 100-bp ladder size marker (right). C. Changes in size of parental macronuclei between 6 h and 28 h after initiation of conjugation. Columns (points) and attached bars correspond to the means of four identical measurements (80-100 cells) and standard deviations.

To understand the functions of *AIF *in *T. thermophila *PND, we knocked out *AIF *by homologous recombination (Figure 4A and 4B). After selection with increasing concentrations of paromomycin, *ΔAIF *strains that did not express AIF were obtained (Figure 4C inset). Similar to the situation in *C. elegans *[16], *ΔAIF *exhibited a somewhat reduced growth rate compared to the wild-type strain (Figure 4C). As shown in Figure 5A, the nuclear events occurring during *Tetrahymena *conjugation could be classified into six stages (A- F). When *ΔAIF *strains of different mating types were mixed, they initiated normal nuclear events (stages A to D; 6-14 h) with the same frequency as the wild-type strain (Figure 5B). The parental macronuclei in the *ΔAIF *strains were reduced in size by 66-85%, whereas those in the wild-type strain were reduced in size by 46-61% at 10-14 h (Figure 5C). These results suggest that AIF is involved in the condensation of the parental macronucleus. At 14-16 h, the *ΔAIF *strain showed a slight delay in transiting from stage C to D (p < 0.01, *t*-test), thereby further implicating the AIF protein in nuclear condensation (Figure 5B). Although the reduction in size of the *ΔAIF *strain occurred after a delay of 4 h, the peak of stage E (Mac IIe) was shifted from 20 h (in the wild-type strain) to 22 h (in the *ΔAIF *strain) (Figure 5B). In addition, the strains exhibited a delay in stage F (Mac III), which corresponds to the final resorption of the parental macronucleus at 20-22 h. Agarose gel electrophoresis revealed that kilobase-size DNA fragmentation occurred in the wild-type strain at 8-10 h (Figure 5B). At this time-point, most of the cells were in stages A to C, suggesting that large-scale DNA fragmentation occurs before nuclear differentiation. However, DNA fragmentation in *ΔAIF *began with a 4-h delay. By 12-14 h, most of the *ΔAIF *cells had undergone nuclear differentiation; however, the reduction in size of the parental macronucleus was also delayed (Figure 5C). Oligonucleosome-size DNA fragmentation was observed in both the wild-type and *ΔAIF *strains 16 h after mating, which suggests that large-scale DNA fragmentation is dependent upon AIF in the early stage of PND (Figure 5B). These results indicate that knocking out *AIF *hinders the first wave of nuclear degradation, including the condensation of the parental macronucleus and kilobase-size DNA fragmentation.

### AIF cooperates with mitochondrial nuclease to promote DNA degradation

**Figure 6:**
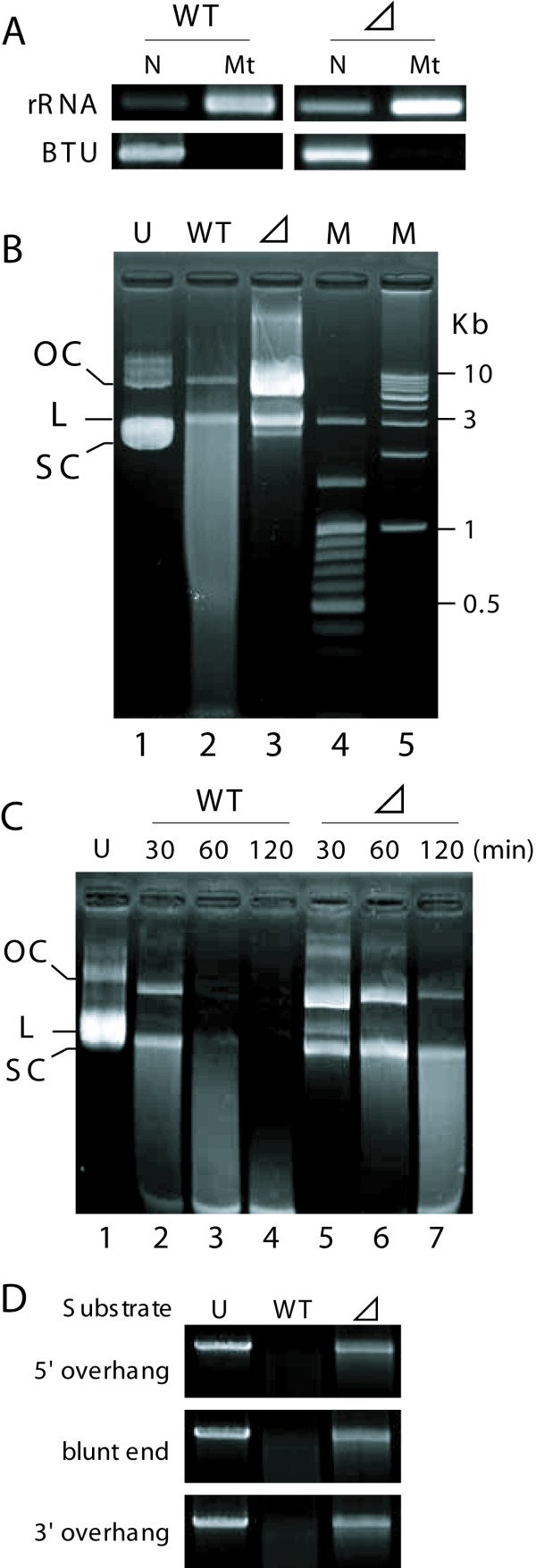
**Mitochondrial nuclease activity**. A. Fractionation PCR. A partial fragment of the mitochondrial large subunit ribosomal RNA (rRNA) or *β-tubulin *(BTU) was amplified by PCR, using fraction samples from wild-type and *ΔAIF *that contained equal amounts of DNA. N and Mt indicate nuclei/unbroken cell fraction and mitochondrial fraction, respectively. No contamination of nuclear DNA was detected in mitochondrial fraction. B. Purified mitochondria (2 μg protein) from wild-type (lane 2) and *ΔAIF *(lane 3) were incubated with 2 μg substrate plasmid DNA with a circular form for 30 min at 37°C in 30 μl reaction buffer containing 20 mM KCl and 50 mM MOPS (pH 6.5). Lane 4 (M) and 5 (M) indicate 100-bp ladder size marker and λHindIII-digest, respectively. The substrate DNA appears in the nicked open circular (OC), linear (L), and supercoiled (SC) forms. C. The nuclease assay was performed under various incubation times. Lane 2-4, substrate DNA was coincubated with wild-type mitochondria. Lane 5-7, substrate DNA was coincubated with *ΔAIF *mitochondria. Undigested sample is seen in lane 1. D. Substrate specificity of the activities. End forms of linear plasmids with 5'- or 3'-overhang or with blunt ends are indicated at the left of gel.

Previously, we demonstrated the presence of strong DNase activity in mitochondria isolated from *Tetrahymena *[7]. To investigate whether the putative mitochondrial DNase of *Tetrahymena *interacts with AIF, mitochondria were purified from vegetatively growing wild-type and *ΔAIF *cells (Figure 6A), and incubated with a circular plasmid as substrate DNA. Mitochondria from the wild-type strain showed strong DNase activities, in that they converted the supercoiled DNA into an open circular form that could be further cleaved into smaller fragments, yielding a smear of degradation products on the gel (Figure 6B, lane 2). In contrast, no smear was observed when mitochondrial extracts from the KO strain were used; instead, only nicking of the plasmid DNA was observed (Figure 6B, lane 3). In addition, a time-course analysis showed that the level of DNase activity was drastically reduced in the KO strain, as compared with the wild-type strain (Figure 6C). These results are similar to those obtained for *C. elegans*, in which an interaction between Wah-1 and Cps-6 was detected [16]. When linear DNA was employed as the substrate, the KO strain showed low DNase activity, whereas wild-type strain digested completely the substrate DNA (Figure 6D). These results indicate that AIF interacts with the mitochondrial DNase to affect not only nicking activity, but also endonuclease activity.

The second *Tetrahymena *homolog of *AIF*, TTHERM_01104910, contains an FAD/NAD binding domain and an oxidoreductase domain that shares 27% identity with TTHERM_006222710. The continuous expression of TTHERM_01104910 was confirmed both in the vegetative phase and during conjugation. This expression pattern was in accordance with that reported in the *Tetrahymena *Gene Expression Database (TGED; http://tged.ihb.ac.cn/). Knocking out the gene did not influence the mitochondrial DNase activity during PND (Additional File 1, 2). Therefore, the second AIF homolog does not appear to be involved in PND.

## Discussion

In unicellular ciliates, the parental cell-derived cytoplasm is taken over by the progeny nucleus after sexual reproduction. Therefore, the development of PND (i.e., the selective elimination of the parental macronucleus) may have been inevitable when the first ciliate established the spatial differentiation of the germinal and somatic nuclei. PND occurs in a limited area of the cytoplasm and is uncoupled from the plasma membrane events associated with PCD (programmed cell death), for example, Fas ligand- Fas receptor binding; however, PND involves mitochondrial apoptotic effectors, such as EndoG-like DNase activity [7]. Thus, ciliates have developed a novel mechanism for executing PND in which part of the intrinsic machinery (i.e., AIF) used for PCD appears to have been adapted for a specialized form of apoptosis.

The primitive mechanism of apoptosis may have been established as a product of the interaction between an ancestral host cell and its endosymbiotic primitive mitochondria [13]. One of the major pathways of apoptosis, the caspase-dependent pathway, appears to have been independently established in animals later during eukaryotic evolution, given that fungi, plants, and protists commonly lack caspase homologs. Caspase-independent pathways function in mammalian and *C. elegans *apoptosis, as evidenced by the finding that apoptosis can occur in the presence of caspase inhibitors [16,20]. AIF, which is assumed to be evolutionarily ancient because it has been identified in various organisms, ranging from protists to animals, is localized within the intermembrane mitochondrial space [13,21]. Disturbances in AIF can delay several major apoptotic events in the nucleus, including nuclear condensation, chromatin digestion, and DNA loss [10,16,17]. These AIF-mediated events resemble those that occur in the early stages of *Tetrahymena *PND.

### Involvement of AIF in PND

In the present study, we provide the first evidence that AIF is involved in *Tetrahymena *PND. AIF translocates from the mitochondria to the parental macronucleus before nuclear differentiation (Figure 3B-D), and interacts with the mitochondrial DNase, thereby triggering the initial DNA degradation by the DNase, the optimal pH of which is about neutral, indicating a role for AIF in the early stage of PND. Taking these observations into consideration, AIF appears to function as a suicide factor in the parental macronucleus. However, the knocking out of the *AIF *gene in the parental macronucleus only slowed by up to 4 h the early stages of PND, including nuclear condensation and kilobase-size DNA fragmentation (Figure 5B and 5C), and did not completely inhibit the progression of PND. Indeed, by the end of conjugation, the AIF-deficient cells were delayed only approximately 1 h, as compared with the wild-type controls (Figure 5B). Is there a mechanism that compensates for the deficiency of AIF, thereby allowing the appropriate execution of the death program? After translocation of AIF into the parental macronucleus, new macronuclei differentiate somewhat later and initiate gene expression immediately. Gene expression from the zygotic macronucleus is indispensable for the completion of the final resorption by autophagy [3]. This delay can be interpreted in different ways. One possibility is that when the *AIF *mRNA is transcribed in the developing macronuclear anlage and the zygotic AIF protein becomes available, the DNA in the parental macronucleus begins to degrade behind schedule, resulting in the recovery of PND progression. It seems most likely that the time lag in gene expression from the zygotic macronucleus is a major cause of the delay in the early stage of PND. Another possibility is that other DNases exist in the *Tetrahymena *mitochondria (E. Osada, personal communication), as identified using SDS-DNA PAGE [22]. Although these DNases are unlikely to either interact with AIF or to be major effectors of PND, they may contribute to the retarded DNA degradation, resulting in the delayed progression of PND.

At the stage of nuclear differentiation, two types of macronucleus, the parental macronucleus and the new zygotic macronucleus, co-exist for a period of time in the same cytoplasm. Through collaboration with the mitochondrial DNase, AIF may prevent simultaneous gene expression from the two different macronuclei with different genotypes through initial digestion of the parental macronuclear DNA.

### Interaction between AIF and the mitochondrial DNase

**Figure 7:**
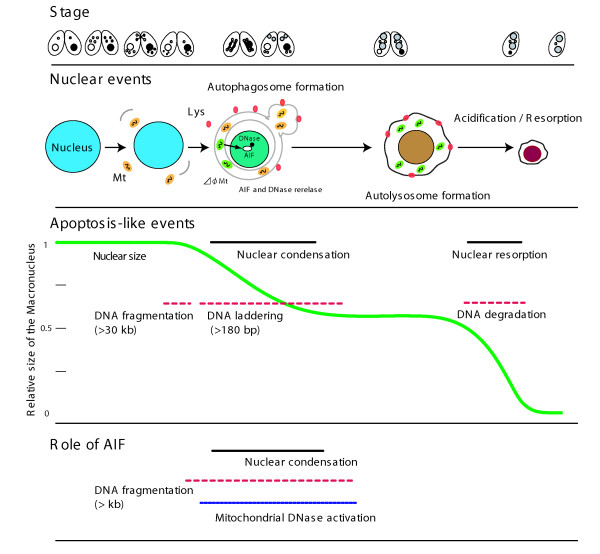
**Schematic representation of PND and a possible role of AIF, based on information described in **[6]**and from the present study**.

In mammals, DNA binding by AIF is required for nuclear condensation and initial DNA cleavage [18]. In contrast, neither FAD/NAD binding ability nor oxidoreductase activity is required for apoptogenesis [11,15]. It remains unclear as to how AIF induces DNA fragmentation during apoptosis. One possibility is that AIF exerts its function by interacting with downstream effectors. AIF and EndoG are two of the many apoptogenic proteins that are released from mitochondria during apoptosis in animals [23]. In *C. elegans*, Wah-1/AIF co-operates with Cps-6/EndoG to promote DNA degradation *in vitro*. In addition, *wah-1 *(*RNAi*) strains and *cps-6 *mutants display similar defects in cell death and DNA degradation, and both Wah-1 and Cps-6 are localized to mitochondria [16]. These findings strongly suggest that the mitochondrial PCD pathway is evolutionarily conserved. Previously, an endonuclease activity was identified in the mitochondria of *T. thermophila *[7]. This activity required divalent cations and was strongly inhibited by Zn^2+^, which is a strong inhibitor of most DNases. In addition, the optimal pH for this endonuclease activity was pH 6.5, while activity was inhibited at pH 5.0, suggesting that the DNase and lysosomal enzymes function in different steps of PND. These characteristics are reminiscent of mammalian mitochondrial EndoG, which mediates the caspase-independent pathway of apoptosis [24]. Indeed, the mammalian EndoG also requires divalent cations, such as Mg^2+ ^and Mn^2+^, exhibits biphasic pH optima of 7.0 and 9.0, and is potently inhibited by Zn^2+ ^[24]. Our plasmid cleavage assay demonstrated that the mitochondria of the *ΔAIF *strains had significantly reduced DNase activity (Figure 6B-D), indicating an interaction between *Tetrahymena *AIF and the DNase. Notably, this result is similar to the aforementioned situation in *C. elegans *[16]. The co-operative action of these two proteins implies that the mitochondrial DNase is an important executor that is activated by AIF. Thus, AIF and mitochondrial DNase appear to constitute a widely conserved DNA degradation pathway that acts in the early stage of apoptosis. However, no sequence homologous to EndoG was detected in the *Tetrahymena *database, raising the possibility that ciliates have independently developed a mitochondrial DNase during the course of ciliate evolution. Indeed, mitochondrial proteomic analysis of *Tetrahymena *has shown that 45% of the proteins are specific to *Tetrahymena *or ciliates [25]. The roles of AIF and mitochondrial DNase are illustrated schematically in Figure 7.

The biochemical and morphologic features of apoptosis have been highly conserved throughout evolution, even in unicellular organisms, such as cellular slime molds, kinetoplastids, dinoflagellates, ciliates, and heterokonts [17,26,27]. A recent study suggested that any protein that has previously been implicated in apoptosis must have a phylogenetically conserved apoptosis-unrelated vital function [28]. For example, AIF serves as a redox-active enzyme in respiratory chain complex I. AIF-deficient mouse embryonic stem cells fail to form a viable embryo [10]. As in *C. elegans*, for which the *wah-1 *(*RNAi*) strains are viable but exhibit a reduced growth rate [16], our *ΔAIF *strains exhibited a slower growth rate, as compared to that of the wild-type strain (Figure 4C). Therefore, AIF may be bifunctional, serving as both a vital protein and a death effector. Among ciliates, apoptosis-like nuclear degradation has been observed during resting cyst formation leading to change in macronuclear DNA content in *Colpoda *[29]. In this case, the macronuclear chromatin is extruded into the cytosol, and the degradation of the extrusion body is accompanied by a reduction in the size of the nucleus, oligonucleosome-size DNA cleavage, and nuclear acidification by lysosomes. This observation indicates that ciliates may have repeatedly adapted their mitochondrial pathway not only for sexual reproduction, but also for cyst formation. Alternatively, it is possible that *Tetrahymena *PND is superficially similar to, but entirely different from animal apoptosis, although AIF participates in this phenomenon. Although no endonuclease G homolog was identified in a survey of the macronuclear genome of *Tetrahymena*, EndoG is present in some other protists, such as the kinetoplastid *Trypanosoma *[30], *Leishmania *[31] and apicomplexan *Cryptosporidium *[32]. In addition, phosphorylation of histone H2AX, which is linked to DNA fragmentation during mammalian apoptosis [33-35], has not been demonstrated in the degenerating macronucleus of *Tetrahymena *[36]. These observations argue for this interpretation, as claimed previously [36]. In conclusion, there are now two incompatible interpretations of the origin of *Tetrahymena *PND: 1) PND developed independently and merely utilized AIF as a suicide factor; and 2) PND shares a common origin with other forms of apoptosis. Identification of the nuclease(s) localized in the mitochondria is needed to elucidate the origin of PND.

## Conclusions

Mitochondrion of *Tetrahymena *contains AIF and yet-unidentified DNase similar to mammalian and *C. elegans *endonuclease G. When new macronuclei are differentiated, AIF translocates from mitochondria to the parental macronucleus in the posterior region of cell. Knockout of AIF showed delayed progression of PND, that is, delay of nuclear condensation and kb-sized DNA fragmentation, corresponding to the initial stage of the nuclear apoptosis. Furthermore, in vitro assay using AIF-deficient mitochondria revealed that mitochondrial DNase acitivity was drastically reduced, suggesting that mitochondrial DNase activity depends upon the presence of AIF. From the results, we presently conclude that mitochondrial AIF might have a major role for executing the nuclear apoptosis of *Tetrahymena *in a simple and primitive fashion, implying its ancient origin.

## Methods

### Culture methods and the induction of conjugation

*T. thermophila *strains CU428 and B2086 (wild-type), were purchased from the National *Tetrahymena *Stock Center (Cornell University). The cells were cultured at room temperature in 2% proteose peptone (Difco), 1% yeast extract (Difco), and 0.5% glucose. To induce mating, the cells were incubated in 0.25% proteose peptone, 0.25% yeast extract, and 4% glucose at room temperature. At mid-log phase, the cells were washed with 10 mM Tris-HCl (pH 7.2) and incubated overnight. To induce conjugation, equal numbers of both strains were mixed and kept at room temperature.

### Cloning of the *T. thermophila *AIF gene and *β-tubulin *promoter

The *T. thermophila *AIF homolog (TTHERM_00622710), including the 1-kb 5'- and 3'-untranslated regions (UTRs), was amplified from CU428 genomic DNA using the following primers: AIF-F (5'-GGTGTTGGTTTGTAGTTC-3') and AIF-R (5'-CACCCAATSGTGAACTGA-3'). Polymerase chain reaction (PCR) was carried out using the following program: 5 min at 94°C followed by 30 cycles of 94°C for 1 min, 46°C for 1 min, and 72°C for 5 min. The resulting 3,966-bp product was cloned into pT7 blue T-vector (Novagen) as a backbone for construction of the knock-out (KO) plasmid. The *β-tubulin *promoter was amplified from CU428 genomic DNA using the following primers: BTU-F-*Not*I (5'-gcggccgcTCCACAGAGACACTAAA-3') and BTU-R-*Eco*RI (5'-gaattcTTTTAATTGCTTAAAGGAGTGA-3'). The PCR program included 5 min at 94°C followed by 30 cycles of 94°C for 1 min, 55°C for 1 min, and 72°C for 1 min. The resulting 809-bp product was cloned into pT7 blue T-vector.

### Cloning of the neomycin resistance gene

The neomycin resistance gene and the *MTT1 *3'-UTR (corresponding to the poly-A signal) were obtained from pTTMN [37]. This region (*Neo*^*r*^) was amplified using the following primers: Neo-F-*Eco*RI (5'-gaattcAAACTTAAAATAATGGCAAG-3') and Neo-R-*Xho*I (5'-ctcgagCCGGGCTGCAGCAATTC-3'). The PCR program included 5 min at 94°C followed by 30 cycles of 94°C for 1 min, 55°C for 1 min, and 72°C for 1 min. The resulting 1,338-bp product was cloned into pT7 blue T-vector.

### Construction of the KO plasmid

Inverse PCR was performed using the *AIF *backbone plasmid as template with the following primers: AIF-F-*Not*I (5'-gcggccgcGTGATTCCTCTTGCGAACAGTTCTT-3') and AIF-R-*Xho*I (5'-ctcgagCTTCTCATCCCGATGT-3'). The start codon of *AIF *was destroyed by changing TAC to GAC in the forward primer. The PCR program included 5 min at 94°C followed by 30 cycles of 94°C for 1 min, 55°C for 1 min, and 72°C for 6 min. The resulting 5,517-bp product was self-ligated and cloned. The plasmid was then digested with *Not*I and *Xho*I and integrated into the *β-tubulin *promoter (*Not*I/*Eco*RI-digested fragment) and *Neo*^*r *^(*Eco*RI/*Xho*I-digested fragment) sites to express *Neo*^*r *^under control of the *β-tubulin *promoter (Neo^r^-cassette). The resultant plasmid (pKoTtAIF) was linearized with *Bam*HI before biolistic bombardment.

### Construction of a GFP-tagged AIF expression plasmid

To obtain the GFP sequence, pTub-tel3 GFP4 [38], which contains codon-optimized *GFP *based on *Paramecium caudatum *codon usage and the *Paramecium *tubulin poly-A signal, was used. This cassette (GFP-cassette) was amplified using the following primers: GFP-F-*Bam*HI (5'-ggatccAGAAAGGGAGAAGAATTGT-3') and GFPpolyA-R (5'-CTCGAGCGGCCGCCAGT-3'). The PCR program included 5 min at 94°C followed by 30 cycles of 94°C for 1 min, 48°C for 1 min, and 72°C for 1 min. The resulting 1,010-bp product was cloned into pT7 blue T-vector. The open reading frame (ORF) of *AIF *and the 1-kb 5'-UTR carrying the *AIF *promoter were amplified from CU428 genomic DNA using the following primers: AIF-F-*Xho*I (5'-ctcgagCACCCAATSGTGAACTGA-3') and AIF-R-*Bam*HI (5'-ggatccAATTTTAGCAGATTAAGAAGC-3'). The PCR program included 5 min at 94°C followed by 30 cycles of 94°C for 1 min, 46°C for 1 min, and 72°C for 3 min. The resulting 3,017-bp product (AIF-cassette) was cloned into pT7 blue T-vector. The backbone of the expression plasmid used in our laboratory contains the *Tetrahymena *telomere sequence and the *Stylonychia *replication origin [39]. This plasmid was digested with *Not*I and *Eco*RI for integration of the Neo^r^-cassette (*Not*I/*Xho*I-digested fragment), AIF-cassette (*Xho*I/*Bam*HI fragment), and GFP-cassette (*Bam*HI/*Eco*RI fragment). The resultant plasmid (pAKgfpTtAIF) was linearized with *Sfi*I before biolistic bombardment.

### *Tetrahymena *transformation

For *Tetrahymena *transformation, mid-log phase cells were harvested by centrifugation and incubated overnight in 10 mM Tris-HCl (pH 7.2). The cells were then centrifuged and packed in 1 ml of 10 mM Tris-HCl at a final concentration of 1 × 10^7 ^cells/ml. A 100- μl aliquot was then spread on a sterile 2-cm circular piece of filter paper. Transformation was achieved using a Biolistic PDS-1000/He Particle Delivery System (Bio-Rad). Gold particles 0.6 μm in size (10 mg/ml in sterile H_2_O) were coated with 5 μg linearized DNA/50 μl particles. Cells were bombarded with the DNA-coated gold particles at 650 psi. Following bombardment, the cells were re-suspended in culture medium and incubated for 6 h. The transformants were screened with 50 μg/ml paromomycin. After three days, the paromomycin-resistant cells were grown in culture medium containing increasing concentrations of paromomycin (from 100 to 1,200 μg/ml) to support the allelic assortment process.

### Reverse transcription (RT)-PCR analysis

Total RNA was extracted from approximately 1 × 10^5 ^cells using Sepasol-RNA1 Super (Nacalai Tesque). Five micrograms of total RNA were used for RT with ReverTra Ace (Toyobo). A 340-bp *AIF*-specific product was produced using the primers AIF-RT-F (5'-AAATCTCTCCACTACACT-3') and AIF-RT-R (5'-AATTTTAGCAGATTAAGAAGC-3'). The program included 1 min at 94°C followed by 30 cycles of 94°C for 1 min, 48°C for 1 min, and 72°C for 30 s.

### Fragmented DNA isolation and agarose gel electrophoresis

Fragmented DNA, such as kb-sized and oligonucleosome-sized DNA, was extracted from the cells at various times during conjugation. In the following procedure, high-molecular-weight DNA is not generally recovered. Cells (1 × 10^5^) were collected by centrifugation (12,000 rpm for 1 min) and re-suspended in cold lysis buffer containing 10 mM EDTA, 0.5% Triton-X 100, and 10 mM Tris-buffer (pH 7.2). After 10 min at 4°C, the lysates were centrifuged at 12,000 rpm for 10 min, and the supernatants were incubated with 0.2 mg/ml RNase for 30 min at 37°C. Proteinase K (0.2 mg/ml) was then added to all samples, which were incubated for 1 h at 37°C. Next, 0.5 M NaCl and 50% 2-propanol were added, and the samples were incubated overnight at -20°C. Fragmented DNA was recovered by centrifugation at 12,000 rpm for 20 min, and the precipitate was dissolved in TAE buffer. Ten micrograms of each DNA sample were then electrophoresed on a 1% agarose gel in TAE and stained with ethidium bromide.

### Indirect immunofluorescence

To image GFP-tagged AIF, cells were fixed in 50% cold methanol and kept on ice for 30 min. After washing with PBS, the cells were blocked in 1% bovine serum albumin (BSA) and incubated for 2 h at room temperature with rabbit polyclonal anti-GFP antibodies (BioReagents) diluted 1:200 in PBS, 1% BSA, and 0.1% Tween20. The cells were washed to remove excess primary antibodies and then incubated with goat anti-rabbit rhodamine-conjugated antibodies (Biomedical Technologies Inc.) for 2 h at room temperature. Excess secondary antibodies were then removed and nuclei were stained with 0.01 μg/μl DAPI for 10 min.

### Preparation of the mitochondria

To isolate mitochondria from wild-type and *AIF*-deficient strains, mid-log phase cells were harvested by centrifugation and washed with 10 mM Tris-HCl (pH 7.2). The washed cell pellets were then re-suspended in cold lysis buffer containing 250 mM sorbitol, 0.2% BSA, 5 mM iodoacetamide, 1 mM EDTA, and 10 mM MOPS-KOH (pH 7.2), and homogenized using Physcotron (Microtec Co., Ltd.) on ice. To remove nuclei and unbroken cells, the lysates were then centrifuged for 5 min at 1,000 × *g*; the supernatants were decanted into Corex centrifuge tubes, followed by centrifugation at 8,000 × *g *for 5 min. Each crude mitochondrial pellet was re-suspended in 500 μl of SEM buffer containing 250 mM sucrose, 1 mM EDTA, and 10 mM MOPS-KOH (pH 7.2). The mitochondria were then purified on discontinuous sucrose gradients consisting of 1.6 M (4 ml) and 1.15 M (7 ml) sucrose in SEM buffer in 13 PET centrifuge tubes. The crude mitochondrial suspensions were layered onto the sucrose gradients and centrifuged at 22,500 rpm for 1 h at 4°C using an RPS40T rotor in an SCP70H ultracentrifuge. The mitochondrial bands were carefully recovered from the interface and transferred into Eppendorf tubes. Mitochondria were collected by centrifugation at 8,000 × *g *for 10 min, the supernatants discarded, and the mitochondrial pellets suspended in SEM buffer. To confirm no contamination of nuclear fraction into mitochondrial fraction, PCR analysis was carried out using specific primers. The promers used are as follows: Mitochondrial large subunit rRNA (mtLSUrRNA) gene; mtLSU-3 (5'-TACAACAGATAGGGACCAA-3') and mtLSU-4 (5'-CCTCCTAAAAAGTAACGG-3'), and *β-tubulin*; BTU-F (5'-TCCACAGAGACACTAAA-3') and BTU-R (5'-ATGCGGTGAGTGCAGAA-3').

### Agarose gel assay for mitochondrial nuclease activity

Substrate plasmid DNA (2 μg of pT7Blue T-vector) was coincubated with isolated mitochondria (2 μg of protein) in 30 μl of reaction buffer containing 20 mM KCl and 50 mM MOPS (pH 6.5) at 37°C. To prepare three types of liner formed DNA which have 3' overhang, blunt-end and 5' overhang, plasmid DNA was digested with *KpnI*, *SmaI *and *BamHI*, respectively, prior to incubation with mitochondria. To quench the reaction, 2% SDS and 10 mM MgCl_2 _were added, and the mixture was incubated at 50°C for 60 min. DNA samples were loaded onto 1.5% agarose gels, electrophoresed, and visualized by staining with ethidium bromide.

## Authors' contributions

TA carried out all experiments and drafted the manuscript. HE critically revised the manuscript and supervised this project. All authors read and approved the final manuscript.

## Supplementary Material

Additional file 1 **Mitochondrial nuclease activity**. A. Purified mitochondria (2 μg protein) from *ΔTTHERM_01104910 *(a: lane 1) and *ΔTTHERM_006222710 *(b: lane 2) were incubated with 2 μg substrate plasmid DNA for 30 min at 37°C in 30 μl reaction buffer containing 20 mM KCl and 50 mM MOPS (pH 6.5). Lane 4 and 5 indicate 100-bp ladder size marker and λHindIII-digest, respectively. The substrate DNA appears in the nicked open circular (OC), linear (L), and supercoiled (SC) forms. B. The nuclease assay was performed under various incubation times. Lane 2-4 (a), substrate DNA was coincubated with *ΔTTHERM_01104910 *mitochondria. Lane 5-7 (b), substrate DNA was coincubated with *ΔTTHERM_006222710 *mitochondria. Lane 1 shows undigested sample. Click here for file

Additional file 2 **Cloning of TTHERM_01104910 and construction of the KO plasmid**. A. One of *AIF *homologs (TTHERM_01104910), including the 1-kb 5' - and 3'-untranslated regions (UTRs), was amplified from CU428 genomic DNA using the following primers: A4910-F (5'-TTACCCTTCACTCAAGCC-3') and A4910-R (5'-ATGGTTGTGCTCGTAGTG-3'). Polymerase chain reaction (PCR) was carried out using the following program: 5 min at 94°C followed by 30 cycles of 94°C for 1 min, 53.5°C for 1 min, and 72°C for 5 min. The resulting 5,281-bp product was cloned into pT7 blue T-vector (Novagen) as a backbone for construction of the knock-out (KO) plasmid. Inverse PCR was performed using the backbone plasmid as template with the following primers: A4910-F-*Not*I (5'-gcggccgcGATCGACTCCAAGAGTCGAA-3') and A4910-R-*Xho*I (5'-ctcgagCTACTTACTTTGCCGC-3'). The start codon of this gene was destroyed by changing TAC to GAC in the forward primer. The PCR program included 5 min at 94°C followed by 30 cycles of 94°C for 1 min, 55°C for 1 min, and 72°C for 8 min. The resulting 7,217-bp product was self-ligated and cloned. The plasmid was then digested with *Not*I and *Xho*I and integrated into the *β-tubulin *promoter (*Not*I/*Eco*RI-digested fragment) and *Neo*^*r *^(*Eco*RI/*Xho*I-digested fragment) sites to express *Neo*^*r *^under control of the *β-tubulin *promoter (Neo^r^-cassette). B. The resultant plasmid (pKoTtA4910) was linearized with *Bam*HI before biolistic bombardment. Click here for file
