# Supplementary material for: Role of apoptosis-inducing factor (AIF) in programmed nuclear death during conjugation in Tetrahymena thermophila
Source: BMC Cell Biol. 2010 Feb 11;11:13. doi: 10.1186/1471-2121-11-13 (PMC2829475; doi:10.1186/1471-2121-11-13)
Supplement: Additional file 1 — Mitochondrial nuclease activity. A. Purified mitochondria (2 μg protein) from ΔTTHERM_01104910 (a: lane 1) and ΔTTHERM_006222710 (b: lane 2) were incubated with 2 μg substrate plasmid DNA for 30 min at 37°C in 30 μl reaction buffer containing 20 mM KCl and 50 mM MOPS (pH 6.5). Lane 4 and 5 indicate 100-bp ladder size marker and λHindIII-digest, respectively. The substrate DNA appears in the nicked open circular (OC), linear (L), and supercoiled (SC) forms. B. The nuclease assay was performed under various incubation times. Lane 2-4 (a), substrate DNA was coincubated with ΔTTHERM_01104910 mitochondria. Lane 5-7 (b), substrate DNA was coincubated with ΔTTHERM_006222710 mitochondria. Lane 1 shows undigested sample. [file 1471-2121-11-13-S1.DOC]

**
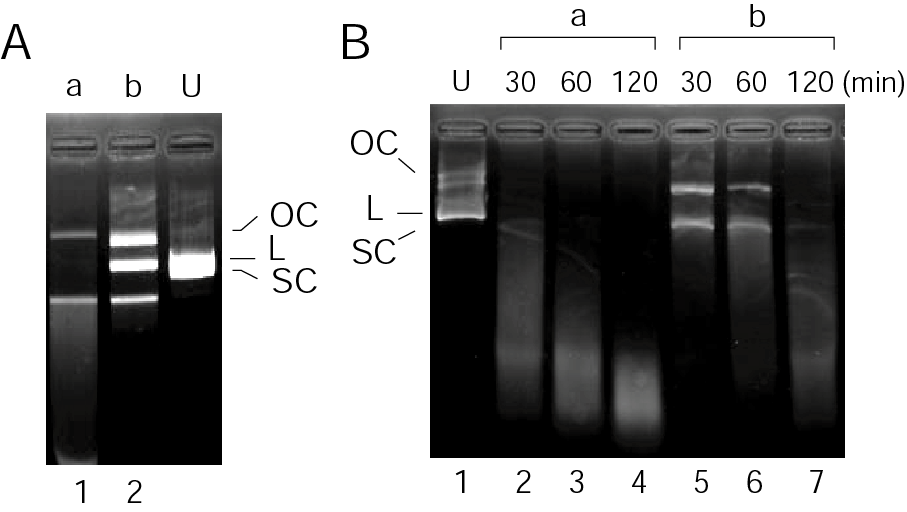
**

**Additional File 1 - Mitochondrial nuclease activity.**

A. Purified mitochondria (2 μg protein) from *ΔTTHERM_01104910* (a: lane 1) and *ΔTTHERM_006222710* (b: lane 2) were incubated with 2 μg substrate plasmid DNA for 30 min at 37℃ in 30μl reaction buffer containing 20 mM KCl and 50 mM MOPS (pH 6.5). Lane 4 and 5 indicate 100-bp ladder size marker and λHindIII-digest, respectively. The substrate DNA appears in the nicked open circular (OC), linear (L), and supercoiled (SC) forms. B. The nuclease assay was performed under various incubation times. Lane 2-4 (a), substrate DNA was coincubated with *ΔTTHERM_01104910* mitochondria. Lane 5-7 (b), substrate DNA was coincubated with *ΔTTHERM_006222710* mitochondria. Lane 1 shows undigested sample.
