# Supplementary material for: Role of apoptosis-inducing factor (AIF) in programmed nuclear death during conjugation in Tetrahymena thermophila
Source: BMC Cell Biol. 2010 Feb 11;11:13. doi: 10.1186/1471-2121-11-13 (PMC2829475; doi:10.1186/1471-2121-11-13)
Supplement: Additional file 2 — Cloning of TTHERM_01104910 and construction of the KO plasmid. A. One of AIF homologs (TTHERM_01104910), including the 1-kb 5' - and 3'-untranslated regions (UTRs), was amplified from CU428 genomic DNA using the following primers: A4910-F (5'-TTACCCTTCACTCAAGCC-3') and A4910-R (5'-ATGGTTGTGCTCGTAGTG-3'). Polymerase chain reaction (PCR) was carried out using the following program: 5 min at 94°C followed by 30 cycles of 94°C for 1 min, 53.5°C for 1 min, and 72°C for 5 min. The resulting 5,281-bp product was cloned into pT7 blue T-vector (Novagen) as a backbone for construction of the knock-out (KO) plasmid. Inverse PCR was performed using the backbone plasmid as template with the following primers: A4910-F-NotI (5'-gcggccgcGATCGACTCCAAGAGTCGAA-3') and A4910-R-XhoI (5'-ctcgagCTACTTACTTTGCCGC-3'). The start codon of this gene was destroyed by changing TAC to GAC in the forward primer. The PCR program included 5 min at 94°C followed by 30 cycles of 94°C for 1 min, 55°C for 1 min, and 72°C for 8 min. The resulting 7,217-bp product was self-ligated and cloned. The plasmid was then digested with NotI and XhoI and integrated into the β-tubulin promoter (NotI/EcoRI-digested fragment) and Neor (EcoRI/XhoI-digested fragment) sites to express Neor under control of the β-tubulin promoter (Neor-cassette). B. The resultant plasmid (pKoTtA4910) was linearized with BamHI before biolistic bombardment. [file 1471-2121-11-13-S2.DOC]

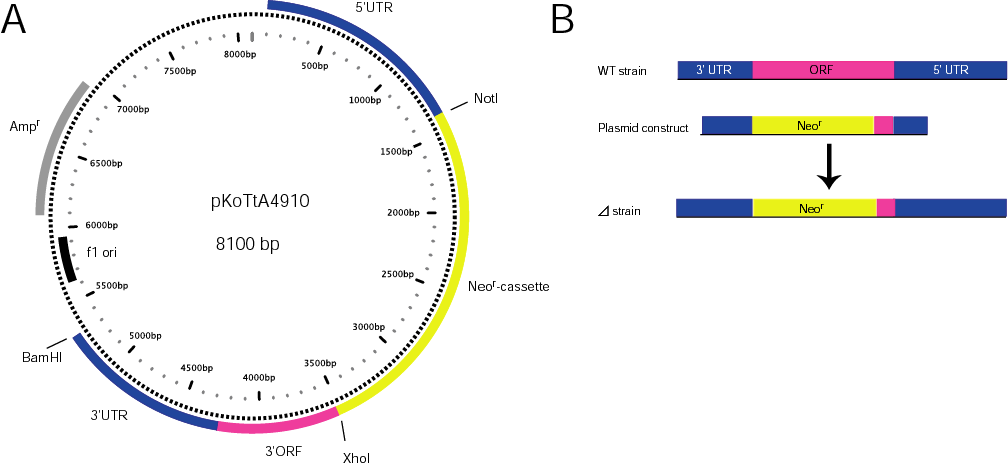


**Additional File 2 - Cloning of TTHERM_01104910 and construction of the KO plasmid.**

A. One of *AIF* homologs (TTHERM_01104910), including the 1-kb 5′- and 3′-untranslated regions (UTRs), was amplified from CU428 genomic DNA using the following primers: A4910-F (5′-TTACCCTTCACTCAAGCC-3′) and A4910-R (5′-ATGGTTGTGCTCGTAGTG-3′). Polymerase chain reaction (PCR) was carried out using the following program: 5 min at 94°C followed by 30 cycles of 94°C for 1 min, 53.5°C for 1 min, and 72°C for 5 min. The resulting 5,281-bp product was cloned into pT7 blue T-vector (Novagen) as a backbone for construction of the knock-out (KO) plasmid. Inverse PCR was performed using the backbone plasmid as template with the following primers: A4910-F-*Not*I (5′-gcggccgcGATCGACTCCAAGAGTCGAA-3′) and A4910-R-*Xho*I (5′-ctcgagCTACTTACTTTGCCGC-3′). The start codon of this gene was destroyed by changing TAC to GAC in the forward primer. The PCR program included 5 min at 94°C followed by 30 cycles of 94°C for 1 min, 55°C for 1 min, and 72°C for 8 min. The resulting 7,217-bp product was self-ligated and cloned. The plasmid was then digested with *Not*I and *Xho*I and integrated into the *β-tubulin* promoter (*Not*I/*Eco*RI-digested fragment) and *Neo^r^* (*Eco*RI/*Xho*I-digested fragment) sites to express *Neo^r^* under control of the *β-tubulin* promoter (Neo^r^-cassette). B. The resultant plasmid (pKoTtA4910) was linearized with *Bam*HI before biolistic bombardment.
